# Association of *GCK* gene DNA methylation with the risk of clopidogrel resistance in acute coronary syndrome patients

**DOI:** 10.1002/jcla.23040

**Published:** 2019-10-11

**Authors:** Jia Su, Nan Zheng, Zhenwei Li, Ning Huangfu, Li Mei, Xiaolei Xu, Li Zhang, Xiaomin Chen

**Affiliations:** ^1^ Department of Cardiology the first Affiliated Hospital Zhejiang University School of Medicine Hangzhou China; ^2^ Department of Cardiology Ningbo Hospital of Zhejiang University Ningbo China; ^3^ Zhejiang University School of Medicine Hangzhou China

**Keywords:** acute coronary syndrome, clopidogrel resistance, DNA methylation, *GCK*

## Abstract

**Backgrounds:**

Clopidogrel resistance (CR), which was manifested as the failure of platelet inhibition in clopidogrel treatment, was likely to lead to cardiovascular events. Our study was aimed to explore the contribution of DNA methylation in glucokinase (*GCK*) to the CR risk.

**Methods:**

Among 36 CR and 36 non‐CR acute coronary syndrome (ACS) patients, the platelet functions were evaluated by VerifyNow P2Y12 assay (turbidimetric‐based optical detection) and DNA methylation levels on two fragments of the CGI from the *GCK* were investigated through bisulfite pyrosequencing methods. In addition, the *GCK* mRNA expression was analyzed via quantitative real‐time PCR. Lastly, the logistic regression was employed to test the interaction between *GCK* methylation and nongenetic variables in CR patients.

**Results:**

Subunit analysis showed that in male patients without DM but suffering from dyslipidemia, the increased methylation of cg18492943 indicated a risk of poor clopidogrel response (male, NCR vs CR(%): 84.86 ± 6.29 vs 88.16 ± 4.32, *P* = .032; without DM, NCR vs CR (%): 84.66 ± 6.18 vs 88.16 ± 4.17, *P* = .029; and dyslipidemia, NCR vs CR (%): 83.81 ± 6.96 vs 88.39 ± 4.74, *P* = .042).In addition, *GCK* mRNA expression was reduced in CR patients without DM. Moreover, regression analysis indicated that the values of platelet distribution width (PDW), total cholesterol (TC), and uric acid (UA) were correlated with the incidence of CR, and hypertension lowered the CR risk.

**Conclusions:**

A higher methylation of cg18492943 in *GCK* gene would lower the expression of *GCK* mRNA, which might contribute to CR in patients without DM. Meanwhile, PDW and TC might be risk factors in CR.

## 1 INTRODUCTION

The dual‐antiplatelet therapy (taking orally aspirin together with ticagrelor or clopidogrel) is used to inhibit platelet activating and aggregating and has been the cornerstone treatment in acute coronary syndrome (ACS) patients after percutaneous coronary intervention (PCI). Although ticagrelor showed fast, consistent, and effective results among ACS patients,^1^ the bleeding incidence was higher than clopidogrel.^2^ Compared with treatment by aspirin and clopidogrel, a latest KAMIR‐NIH research showed that aspirin together with ticagrelor or prasugrel displayed a similar risk of all‐cause mortality but an elevating bleeding risk.^3^ Hence, clopidogrel might be a better treatment in East Asian ACS patients.

Clopidogrel, by inhibiting *P2Y12* receptor, could prevent adenosine diphosphate‐induced platelets aggregation and lower the incidence of major adverse cardiovascular events (MACE) in ACS sufferers after PCI.^4^ While different patients responded greatly to clopidogrel treatment.^5^ Approximately 10%‐40% of patients continue to manifest adverse cardiovascular risk.^6^ This effect may be due to the failure of platelet inhibition responding to clopidogrel, a clinical phenomenon named clopidogrel resistance (CR).^7^ Moreover, by VerifyNow P2Y12 assay, some researchers have noted that P2Y12 reaction units (PRU) higher than 240 could indicate the existence of CR.^8^ But, the CR pathogenesis remains not clearly understood. Many extrinsic factors could influence platelet activity after taking clopidogrel, such as comorbidities, drug interactions, and smoking.^9^ Among these factors, diabetes mellitus (DM) may be an important clinical factor attributed to platelet dysfunction.^10^ The mechanisms involved in hyperreactive platelets in DM patients have been suggested to be insulin deficiency, hyperglycemia, metabolic conditions, and cellular abnormalities.^11^

The glucokinase gene (*GCK*), sited on 7p15.3‐p15.1, codes the glucose enzyme.^12^ Through catalyzing the rate‐limiting step of insulin secretion, *GCK* participates in balancing glucose homeostasis and glycogen synthesis.^13^ Many studies have indicated that *GCK* plays a vital role in diabetes. Through systemic analysis of gene expression profiles, one study revealed that the *GCK* gene played a role in obese type 2 diabetes.^14^ Another study investigated the effect of DNA methylation on obesity and type 2 diabetes mellitus and found that DNA hypermethylation could lead to a decline in hepatic *GCK* expression and was correlated with susceptibility to diabetes.^15^ Additionally, if *GCK* DNA methylation changes, then glucose homeostasis in F1 offspring would be disrupted after maternal bisphenol A exposure.^16^ Although our former studies showed that the DNA methylation of P2Y12^17^ and PON1^18^ might be associated with CR, the effect of *GCK* DNA methylation on CR is not clearly understood. In this study, in ACS patients with clopidogrel treatment, we attempted to explore whether the selected CpG islands methylation in *GCK* is associated with CR.

## 2 METHOD

### 2.1 Samples

Seventy‐two ACS patients were picked up in Ningbo No. 1 Hospital from September 2012 to December 2018. These patients, who complained of chest pain, were diagnosed by physical examination, laboratory tests, and coronary angiography. The followings were our inclusion criteria: (a) Based on recent ESC guideline, these ACS patients underwent PCI through drug‐eluting stent; (b) before PCI, the patients received 300 mg clopidogrel and 300 mg aspirin as loading doses, and they were subsequently administered 75 mg clopidogrel and 100 mg aspirin daily as a maintenance doses; and (c) the patients were without aspirin low response (aspirin reaction units <550). And our exclusion criteria were shown as below: (a) chronic heart failure; (b) acute infection; (c) rheumatological disorders; (d) history of malignancy; (e) history of active bleeding; (f) abnormal hepatic or kidney function; (g) concomitant treatment using warfarin or IIb/IIIa inhibitors (Tirofiban); and (h) platelets <150 000 μL or more than 500 000 μL.

Ethical approval was obtained for human sample collection from the Ethics Committees at Ningbo No. 1 Hospital, and all selected patients offered their written informed consent. The study protocol conformed to the principles outlined in the Declaration of Helsinki.

### 2.2 Biochemical analyses and platelet function measurements

Blood samples from 36 CR and 36 NCR patients were collected, and their biochemical values, such as LDL, GLU, HbA1c, and BUN, were tested based on a standard process according to the manufacturer, and the raw data were collected.

The 2016 ACC/AHA guideline^19^ pointed out that the ACS patients, who would more likely to suffer the risk of CVD after PCI, were suggested to access platelet function. Thus, in this study, the patient platelet function was measured after 1 month since PCI. Through VerifyNow P2Y12 assay (Accumetrics Inc), platelet function was assessed to evaluate the responsiveness to P2Y12 antagonists.^20^ This turbidimetric‐based optical detection system evaluated platelet aggregation as an increase in light transmittance from whole blood. The result was informed as PRU. And the PRU greater than 240 indicated the existence of clopidogrel resistance.^8^

### 2.3 DNA extraction and methylation assay

Human genomic DNA was extracted from leukocytes via the DNA Blood Mini Kit (Qiagen). DNA concentrations were determined by NANODROP 1000 (Nanodrop) to ensure more than 500 ng/µL *GCK* DNA methylation of cg01700200 and cg18492943 were assessed by bisulfite pyrosequencing. First, sodium bisulfite DNA conversion was carried out by EpiTech Bisulfite Kits. Then, polymerase chain reaction (PCR) amplification was operated using the PyroMark PCR Kit. Finally, the targeted CpG islands methylation was sequenced through PyroMark Qiagen Q96 reagents. Based on PyroMark Assay Design software, PCR primers and pyrosequencing primers were automatically planned, as shown in Table 1.

**Table 1:** Primers for cg01700200 and cg18492943 CpG island loci analysis

	Group	DNA sequence (5′ → 3′)	Modification	Purification
cg01700200	Forward primer	AGTGATAGGTAGATTTGGGATTT		PAGE
	Reverse primer	AAAAAAAAAACAACCAACCCAAATA	5'Biotin	HPLC
	Sequencing primer	ACCAACCCAAATAAAAAAAT		PAGE
cg18492943	Forward primer	GGTTATAGGAATGTTGAAGAATGTAG	5'Biotin	PAGE
	Reverse primer	ACACTTACAACAACCATAAAAATACT		HPLC
	Sequencing primer	ACAACAACCATAAAAATACTC		PAGE

### 2.4 Level of *GCK* mRNA determination

Using a RNeasy Plus Universal Kit (QIAGEN), RNA was separated from blood samples. One microgram of RNA along with the PrimeScript^™^ RT Reagent Kit and gDNA Eraser (Takara Bio) was used to generate cDNA. Then, after diluting the template cDNA, the relative expression of *GCK* mRNA was measured by the ABI 7500 qRT‐PCR System (Applied Biosystems). GAPDH was applied to normalize *GCK* expression. The relative quantitative was implemented via the comparative CT method (ΔΔCt). The amplification primers involving in qRT‐PCR were schemed by Prime 5 software, and the sequences are shown in Table 2.

**Table 2:** Primers for *GCK* mRNA analysis

Name	Group	Base sequence (5′ → 3′)
PON1	Forward primer	GAGGCCGCCAAGAAGGAGAA
	Reverse primer	GTAGTCGAAGAGCATCTCA
GADH	Forward primer	GGACCTGACCTGCCGTCTA
	Reverse primer	AGGAGTGGGTGTCGCTGT

### 2.5 Statistical analysis

Statistical breakdown was operated with the software of PASW 18.0 (SPSS, Inc). A collection of association analyses were performed between mRNA expression, *GCK* DNA methylation, clinical indexes, and CR. Continuous variables that were not normally distributed are be described as medians with interquartile ranges (IQRs) and compared with non‐parametric test, and normally distributed values are shown as the mean with standard deviation and compared by *t* test. Additionally, categorical variables are expressed as the mean ± standard deviation and were evaluated by either chi‐square tests or Fisher's exact tests. The *GCK* mRNA expression levels are shown as the median with IQRs and compared with the *t* test. Multiple linear regression was used to determine the effect between *GCK* DNA methylation and clinical features. Logistic regression was utilized for analyzing correlations between *GCK* methylation and biochemical elements among ACS patients suffered from clopidogrel resistance. A two‐sided *P* value, which was < .05, was regarded as statistically significant.

## 3 RESULTS

### 3.1 Patient characteristics

From May 2010 to December 2018, a total of 72 CAD patients who met the above requirements were recruited in this study. Among these patients, 36 were considered CR. The clinical baselines of CR and NCR patients are presented in Table 3. Except for uric acid (UA), the other clinical indexes were well matched. This result indicated that higher uric acid might increase the CR risk.

**Table 3:** The comparison of characteristics between CR and NCR patients

Index	NCR (n = 36)	CR (n = 36)	*χ^2^/z/t*	*P* value
Age (y)	62.7 ± 10.1	64.2 ± 10.7	−0.656	.514
BMI (kg/m^2^)	23.65 ± 2.49	24.04 ± 3.11	0.183	.560
Left ventricular ejection fraction (%)	62.3 ± 6.5	58.0 ± 11.1	2.125	.037
Total cholesterol (mg/dL)	4.333 ± 1.176	4.863 ± 1.344	−1.778	.080
Triglyceride (mg/dL)	1.475 ± 1.043	1.741 ± 1.108	−1.046	.299
HDL (mg/dL)	1.020 ± 0.253	0.939 ± 0.246	1.376	.173
LDL (mg/dL)	2.610 ± 1.020	2.908 ± 1.052	−1.221	.226
Albumin (g/L)	38.98 ± 4.14	38.01 ± 5.32	0.852	.397
Blood sugar	5.561 ± 1.242	6.211 ± 2.002	−1.653	.103
HbA1c (%)	6.06 ± 0.97	6.48 ± 1.40	−1.476	.144
ALT (umol/L)	33.3 ± 26.6	39.1 ± 24.7	−0.798	.428
AST (umol/L)	26.0 (18.0,81.5)	28.0 (18.5,115.3)	−0.180	.857
TBIL (mmol/L)	14.49 6.40	13.95 9.95	0.276	.783
BUN (mmol/L)	5.423 1.305	6.232 2.886	−1.534	.130
Cr (μmol/L)	72.69 ± 18.48	73.59 ± 23.16	−0.182	.856
UA (ummol/L)	267.0 ± 164.2	341.6 ± 86.9	−2.408	.019
HsCRP (mg/dL）	2.0 (1.1,7.7)	5.2(4.7,6.3)	−0.265	.791
PLT (10^9^/L)	205.9 ± 74.7	184.0 ± 63.5	1.338	.185
MPV (fL)	8.383 ± 1.244	8.433 ± 1.504	−0.154	.878
PCT (%)	0.173 ± 0.054	0.152 ± 0.042	1.791	.878
PDW (fL)	15.54 ± 1.98	16.4 ± 0.61	−2.665	.011
Male Gender	26 (71.2)	26 (71.2)	‐	‐
Alcohol abuse	10 (27.8)	6 (16.7)	1.286	.257
Hypertension, n (%)	24 (66.7)	25 (69.4)	0.064	.800
Diabetes, n (%)	7 (19.4)	11 (30.6)	1.185	.276
Dyslipidemia, n (%)	16 (44.4)	15 (41.7)	0.057	.812
Current smoking, n (%)	11 (30.6)	13 (36.1)	0.250	.617

### 3.2 The association of CR and *GCK* DNA methylation

**Figure 1:**
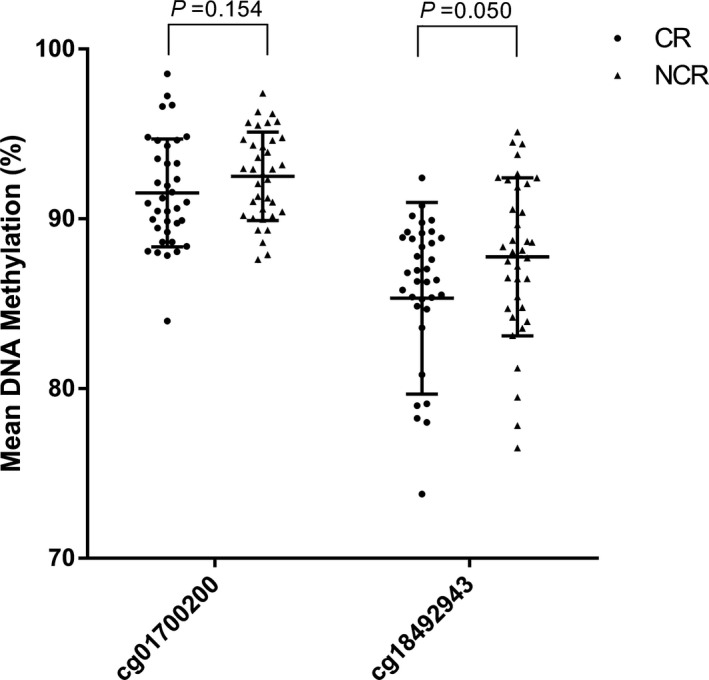
The comparison of cg01700200 and cg18492943 DNA methylation in *GCK* between CR and NCR patients

In the present research, according to the reference of our 850 k methylation chip, we chosecg01700200 (chr7:44227705‐44227975) and cg18492943 (chr7:44203220‐44203435) on *GCK* for bisulfite pyrosequencing. All of the genes were located on the gene body. We evaluated the association of the *GCK* DNA methylation between CR and NCR patients. As displayed in Figure 1 and Table 4, in targeted fragments, the methylation levels in cg01700200 and cg18492943 were not significantly interrelated with clopidogrel poor response.

**Table 4:** The comparison of GCK gene DNA methylation levels between CR and NCR patients

Meth. (%)	NCR	CR	*t*	*P* value
cg01700200	92.51 ± 2.60	91.53 ± 3.17	1.439	.154
cg18492943	85.33 ± 5.65	87.76 ± 4.65	−1.995	.050

**Figure 2:**
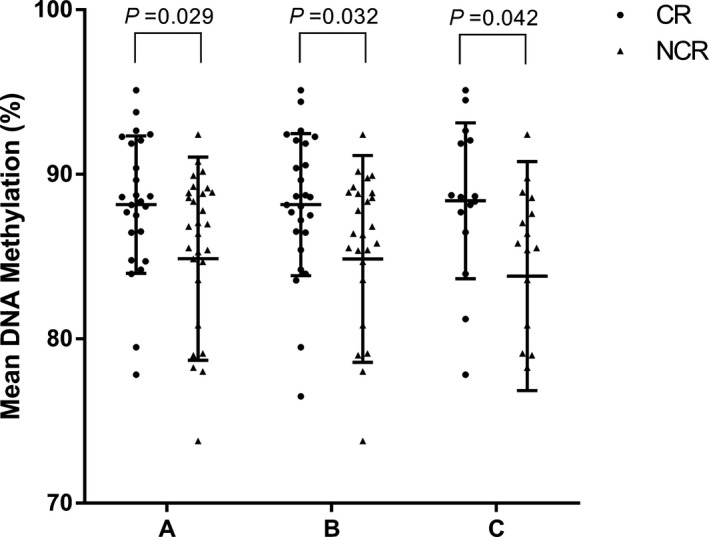
The comparison of cg01700200 and cg18492943 DNA methylation in *GCK* between CR and NCR patients in the subgroups. (A, male patients, NCR vs CR (%): 84.86 ± 6.29 vs 88.16 ± 4.32, *P* = .032; B, patients without DM, NCR vs CR (%): 84.66 ± 6.18 vs 88.16 ± 4.17, *P* = .029; and C, patients with dyslipidemia, NCR vs CR (%): 83.81 ± 6.96 vs 88.39 ± 4.74, *P* = .042)

Then, by different clinical baseline characteristics, we carried out a subunit analysis to estimate whether the *GCK* DNA methylation (cg01700200 and cg18492943) were associated with CR. We observed that in male patients without DM but suffering from dyslipidemia, higher methylation of cg18492943 indicated a risk of poorer clopidogrel response (male, NCR vs CR (%): 84.86 ± 6.29 vs 88.16 ± 4.32, *P* = .032; without DM, NCR vs CR (%): 84.66 ± 6.18 vs 88.16 ± 4.17, *P* = .029; and Dyslipidemia, NCR vs CR (%): 83.81 ± 6.96 vs 88.39 ± 4.74, *P* = .042). However, our study did not discover any significant relations between cg01700200 DNA methylation and poor response to clopidogrel, and there were no meaningful findings in other subgroups (Figure 2 and Table 5).

**Table 5:** The comparison of GCK gene DNA methylation levels between CR and NCR patients in the subgroups

Index	Meth. (%)	NCR	CR	*t*	*P* value
DM					
Yes (7 vs 11)	cg01700200	91.15 ± 2.63	91.71 ± 2.73	−0.581	.569
	cg18492943	87.20 ± 1.72	86.85 ± 5.72	0.155	.879
No (29 vs 25)	cg01700200	92.84 ± 2.53	91.36 ± 3.39	1.834	.072
	cg18492943	84.66 ± 6.18	88.16 ± 4.17	−2.252	.029
Male					
Yes (26 vs 26)	cg01700200	92.63 ± 2.53	91.70 ± 3.37	1.136	.261
	cg18492943	84.86 ± 6.29	88.16 ± 4.32	−2.208	.032
No (10 vs 10)	cg01700200	92.17 ± 2.88	91.07 ± 2.68	0.883	.389
	cg18492943	86.55 ± 3.45	86.72 ± 5.54	−0.083	.935
Hypertension					
Yes (24 vs 25)	cg01700200	92.74 ± 2.78	91.22 ± 3.23	1.766	.084
	cg18492943	85.09 ± 6.51	87.44 ± 5.19	−1.404	.167
No (12 vs 11)	cg01700200	92.05 ± 2.25	92.22 ± 3.07	0.218	.877
	cg18492943	85.81 ± 3.55	88.49 ± 3.19	0.792	.072
Dyslipidemia					
Yes (16 vs 15)	cg01700200	92.27 ± 2.38	90.95 ± 3.99	0.047	.270
	cg18492943	83.81 ± 6.96	88.39 ± 4.74	−0.296	.042
No (20 vs 21)	cg01700200	92.71 ± 2.81	91.94 ± 2.45	0.384	.357
	cg18492943	86.54 ± 4.13	87.31 ± 4.65	0.330	.578
Hyperuricemia					
Yes (7 vs 14)	cg01700200	93.69 ± 3.56	91.64 ± 4.16	1.131	.313
	cg18492943	85.03 ± 10.98	85.41 ± 5.54	−0.109	.090
No (29 vs 22)	cg01700200	92.41 ± 2.60	91.21 ± 2.00	1.801	.095
	cg18492943	86.92 ± 3.12	87.24 ± 5.06	−0.178	.060
Current smoking					
Yes (11 vs 13)	cg01700200	92.47 ± 2.45	91.84 ± 4.15	0.194	.667
	cg18492943	85.85 ± 4.83	87.12 ± 4.56	0.963	.341
No (25 vs 23)	cg01700200	92.53 ± 2.71	91.35 ± 2.56	0.782	.128
	cg18492943	85.10 ± 6.05	87.79 ± 4.80	0.870	.097
Alcohol abuse					
Yes (10 vs 6)	cg01700200	92.64 ± 2.29	92.13 ± 2.52	0.923	.686
	cg18492943	84.71 ± 5.29	89.36 ± 5.34	0.834	.112
No (26 vs 30)	cg01700200	92.46 ± 2.75	91.41 ± 3.31	0.492	.204
	cg18492943	85.57 ± 5.86	87.44 ± 4.53	0.826	.183

### 3.3 The association of *GCK* mRNA expression and clopidogrel resistance

**Figure 3:**
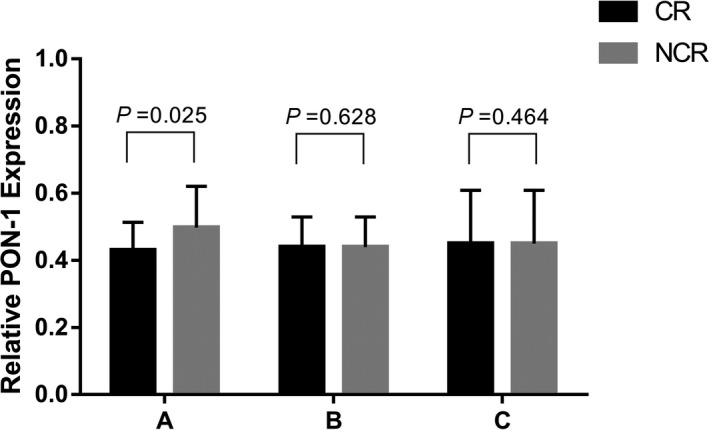
The comparison of *GCK* mRNA expression between CR and NCR patients in the subgroups. (A, male patients; B, patients without DM; and C, patients with dyslipidemia)

Through qRT‐PCR, we assessed the relative mRNA expression in *GCK* to determine that the various *GCK* expression levels would influence on clopidogrel response. Unfortunately, the result was insignificant. While in a subgroup of patients without DM, we observed that *GCK* mRNA expression was decreased when patients were suffering from clopidogrel poor response (Figure 3).

### 3.4 Regression analysis

For various clinical factors that might influence DNA methylation, we investigated the effect of these variables on *GCK* gene methylation through multiple linear regression, and we discovered that uric acid might be related to the methylation of cg18492943 (*F* = 1.092, *P* value = 0.041, *R* ^2^ = 0.300). Nevertheless, other confounding factors did not affect the methylation of cg01700200 and cg18492943.

Moreover, in consideration of genetic and nongenetic factors influenced on clopidogrel response, we implemented a logistic regression analysis to assess the association between CR and these confounding factors. The results revealed that these variables (such as cg18492943, age, AST, TC, and PDW) were correlated with CR, while hypertension was protective factors of CR (Table 6).

**Table 6:** Logistic regression analysis in CR and NCR patients

	B	Std. Error	Wald	*P* value	Exp (B; 95% C.I)
cg18492943	0.128	0.066	3.773	.052	1.137 (0.999,1.293)
Age	0.059	0.034	3.016	.082	1.060 (0.992,1.133)
TC	1.117	0.371	9.058	.003	0.327 (0.158,0.677)
T	0.007	0.003	7.585	.006	1.008 (1.002,1.013)
PDW	1.108	0.429	6.663	.010	3.030 (1.306,7.029)
Hypertension	−1.425	0.712	4.010	.045	0.241 (0.060,0.970)
Constant	−27.943	9.666	8.357	.004	0.000

Furthermore, since we performed logistic regression analysis in patients without DM, male, and dyslipidemia subgroups, the results indicated that in patients without DM, cg18492943, TG, TC, BUN, and PDW showed the risk of poor clopidogrel response, while cg01700200 was a protective factor (Table 7). However, the logistic regression analysis in other subgroups did not show any significant outcomes.

**Table 7:** Logistic regression analysis in the subgroup without DM

	B	Std. Error	Wald	*P* value	Exp (B) (95% C.I)
cg01700200	−0.338	0.150	5.108	.024	0.713 (0.532,0.956)
cg18492943	0.275	0.129	4.522	.033	1.317 (1.022,1.698)
TC	0.687	0.351	3.843	.050	0.503 (0.253,1.000)
TG	2.096	0.831	6.361	.012	8.130 (1.595,41.434)
BUN	0.401	0.262	2.341	.126	1.493 (0.894,2.494)
PDW	1.084	0.617	3.084	.079	2.956 (0.882,9.908)
Constant	−12.610	19.449	0.420	.517	0.000

## 4 DISCUSSION

Antiplatelet medicines (aspirin or clopidogrel) are commonly prescribed to reduce cardiovascular risk, especially in ACS patients after PCI. Patients taking antiplatelet drugs still experience bleeding or ischemic events, which represents a significant public health issue. Although new P2Y12 inhibitors, such as ticagrelor, could more rapidly, consistently, and effectively inhibit platelet activity,^1^ these drugs cause more bleeding events.^2^ A lately COSTIC research presented on 2018 ESC noted that clopidogrel was more suitable for Chinese. Hence, more investigations concerning the different responses to clopidogrel treatment, and personalized approaches to antiplatelet therapy in Chinese ACS patients are increasingly being considered.^21^ Since we carried out personalized approaches in antiplatelet treatment in clinical practise, we should note the existence of CR. A large number of studies have investigated the underlying mechanisms, and novel molecular mechanisms are continuously being discovered. For example, genetic polymorphisms (ABCB1,^22^, ^23^ CYP2C19, and PON1^24^) sometimes accounted for the risk of CR. However, polymorphisms failed to explain the gene‐environment interaction, and accumulating evidence suggests that DNA methylation modification, which occurs at cytosine‐phosphate‐guanine (CpG) dinucleotide sites, is a reliable and stable epigenetic modification^25^ and can actively reshape pathological processes. Usually, the hypermethylation of CpG islands (CGIs) might induce transcriptional silencing and influence the expression of targeted proteins. A recent investigation reported that aberrant DNA methylation might participate in the occurrence and development of atherosclerotic plaques^26^ and coronary heart disease.^27^ Furthermore, some studies have demonstrated that changes in ABCB1,^28^ ABCC3,^29^ P2Y12,^17^ and PON1^18^ promoter methylation levels were associated with a decline in clopidogrel antiplatelet effects.

In the present study, according to the latest reference of our 850 k methylation chip (SUPPORTING INFORMATION), we found that in male patients without DM but suffering from dyslipidemia, higher cg18492943 methylation of the *GCK* gene indicated the occurrence of CR. In patients without DM, the expression of *GCK* mRNA was lower. Previous studies have also focused on the effect of *GCK* gene methylation on different diseases and physical disorders. Some studies discovered that hypomethylation in the *GCK* gene body was correlated to a risk of coronary heart disease^30^ and essential hypertension.^31^ However, few studies have paid close attention to the relationship between CR and *GCK* DNA methylation. Our recent results indicated that higher DNA methylation at cg18492943 could lead to lower the expression of GCK mRNA, which might contribute to CR in patients without DM. This effect might be due to glucostasis and metabolism. As this protein is predominantly produced in the pancreas, *GCK* catalyzes insulin secretion and participates in glycogen synthesis.^13^ The lower expression of the *GCK* gene would induce abnormal insulin secretion, leading to glucose instability. One study reported that elevated methylation in *GCK* CpG4 suggested higher risk for type 2 diabetes in Chinese males.^32^ DM patients exhibited an impaired response to clopidogrel, attributable to the attenuation of the PK profile of clopidogrel and an altered functional status of the P2Y12 signaling pathway.^33^ Hence, diabetes mellitus or pathoglycaemia was one of the major independent predictive factors for clopidogrel non‐responsiveness.^34^ In our present cohort, for non‐DM patients, the hypermethylation of cg18492943 on *GCK* might interfere with glucose stability, leading to the occurrence of CR. However, sample size limited our further evaluation and functional experiments on *GCK* methylation, such as cellular validation, are needed to further elucidate the inherent mechanism.

Except for the *GCK* gene, other aspects might also influence glucose regulation and the response to clopidogrel.^11^ Among these aspects, deficient insulin action is one of the most cardinal factors contributing to platelet dysfunction.^35^ Platelets express insulin receptors and insulin‐like growth factor‐1 (IGF‐1) receptors.^36^ IGF‐1 is present in granules of platelets, and its functional receptors are expressed on the platelet surface, leading to the amplification of platelet responses. Moreover, IGF‐1 stimulation contributes to the phosphorylation of the IGF receptor and the tyrosine phosphorylation of insulin receptor substrate‐1 (IRS‐1) and IRS‐2. The latter subsequently bound to the p85 subunit of phosphoinositide‐3 kinase (PI3K), resulting in phosphorylation of the protein kinase B, which participated in several cellular responses to insulin as well as IGF‐1 and was involved in the modulation of the platelet response.^37^ In addition, an increase in intracellular calcium promotes platelet degranulation and aggregation. IRS‐1 may mediate the inhibition of Ca^2+^ mobilization through insulin.^38^ In T2DM patients, SNPs of rs956115 and rs13431554 in IRS‐1 have hyperreactive platelets and suffer from a risk of ischemic events.^39^ IRS‐independent pathways contribute to platelet hyperreactivity due to impaired responses to nitric oxide (NO) and prostacyclin, which are related to CR.^40^ In our study, *GCK*, influenced by DNA methylation, could affect platelet activity in response to clopidogrel, which was also involved in insulin deficiency. However, the exact mechanisms of insulin action and CR have not been well established, and future discoveries will help us to understand the possible molecular factors and signaling pathways.

In addition to genetic factors, various extrinsic elements (drug interactions, comorbidities, and environment) may also result in CR. Our subgroup analysis and logistic regression analysis in present study discovered that hypertension decreased the CR risk, and cg18492943, PDW, TC, and UA may be positively associated with clopidogrel resistance. First, platelet distribution width (PDW) could manifest morphological changes in platelets, and platelet size was suggested as an indicator of enhanced reactivity; thus, the higher PDW might potentially increase the risk of complications after stenting.^41^ Another study demonstrated that the ratio of platelet to red cell distribution width (P‑RDW) was lower in high on‑treatment platelet reactivity (HPR) patients.^42^ As a result, an assessment of the role of PDW in planning antiplatelet therapy is warranted. Second, we discovered that total cholesterol was a protective factor. This finding was similar to a recent study, which also reported that a poorer clopidogrel response was significantly associated with levels of total cholesterol.^43^ Third, for the factors of uric acid and hypertension, the conclusions were not consistent. A previous correlation analysis revealed that hypertension and uric acid were risk factors for clopidogrel resistance,^44^ but another study showed that uric acid levels did not influence poor responses to clopidogrel.^45^ A systemic meta‐analysis and larger sample trials would provide reliable conclusions.

To our knowledge, it was the first research exploring the association of *GCK* DNA methylation along with its mRNA relative expression with clopidogrel resistance. During this study, numerous attempts were made, but several inherent limitations were existed. First, we selected two fragments of the CGI from *GCK*, and there are likely other regions related to CR. Future analyses would help us to further our exploration. Second, based on animal cells or levels, functional experiments on *GCK* methylation are needed to validate the inherent mechanism in CR. Third, as gene‐environment and gene‐gene reciprocal interactions exist, unidentified confounding factors might change the expression *GCK* of genes and induce biased results. However, it is worthwhile to explore the molecular mechanism of clopidogrel resistance. Multicentre studies with large samples would optimize our discovery and further assessment.

To sum up, our study suggested that the higher methylation of cg18492943 in *GCK* gene could lead to a decline in *GCK* mRNA expression and potentially cause CR in patients without DM. Additionally, subgroup analysis and logistic regression analysis showed that the values of PDW, TC, and UA were interrelated with the incidence of CR, and hypertension lowered the CR risk. However, other advanced and effective planning studies with larger samples would facilitate the validity of our findings and the elucidation of CR pathogenesis.
