# Supplementary figures and images for: Association of GCK gene DNA methylation with the risk of clopidogrel resistance in acute coronary syndrome patients
Source: J Clin Lab Anal. 2019 Oct 11;34(2):e23040. doi: 10.1002/jcla.23040 (PMC7031555; doi:10.1002/jcla.23040)

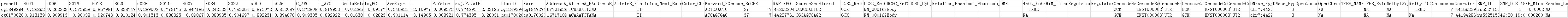

Supplement: Supplementary file 1 [file JCLA-34-e23040-s001.tif]
